# Providing the Best Parenteral Nutrition before and after Surgery for NEC: Macro and Micronutrients Intakes

**DOI:** 10.3390/nu14050919

**Published:** 2022-02-22

**Authors:** Silvia Guiducci, Miriam Duci, Laura Moschino, Marta Meneghelli, Francesco Fascetti Leon, Luca Bonadies, Maria Elena Cavicchiolo, Giovanna Verlato

**Affiliations:** 1 Neonatal Intensive Care Unit, Department of Women’s and Children’s Health, University Hospital of Padova, 35128 Padova, Italy; silviaguiducci24@gmail.com (S.G.); lauramoschino13@gmail.com (L.M.); meneghelli.marta@gmail.com (M.M.); luca.bonadies@aopd.veneto.it (L.B.); mariaelena.cavicchiolo@aopd.veneto.it (M.E.C.); 2 Pediatric Surgery Unit, Department of Women’s and Children’s Health, University Hospital of Padova, 35128 Padova, Italy; ducimiriam@gmail.com (M.D.); francesco.fascettileon@unipd.it (F.F.L.)

**Keywords:** necrotizing enterocolitis, surgery, parenteral nutrition, macronutrients, micronutrients, parenteral nutrition associated liver disease

## Abstract

Necrotizing enterocolitis (NEC) is the main gastrointestinal emergency of preterm infants for whom bowel rest and parenteral nutrition (PN) is essential. Despite the improvements in neonatal care, the incidence of NEC remains high (11% in preterm newborns with a birth weight <1500 g) and up to 20–50% of cases still require surgery. In this narrative review, we report how to optimize PN in severe NEC requiring surgery. PN should begin as soon as possible in the acute phase: close fluid monitoring is advocated to maintain volemia, however fluid overload and electrolytes abnormalities should be prevented. Macronutrients intake (protein, glucose, and lipids) should be adequately guaranteed and is essential in each phase of the disease. Composite lipid emulsion should be the first choice to reduce the risk of parenteral nutrition associated liver disease (PNALD). Vitamin and trace elements deficiency or overload are frequent in long-term PN, therefore careful monitoring should be planned starting from the recovery phase to adjust their parenteral intake. Neonatologists must be aware of the role of nutrition especially in patients requiring long-term PN to sustain growth, limiting possible adverse effects and long-term deficiencies.

## 1. Introduction

**Figure 1:**
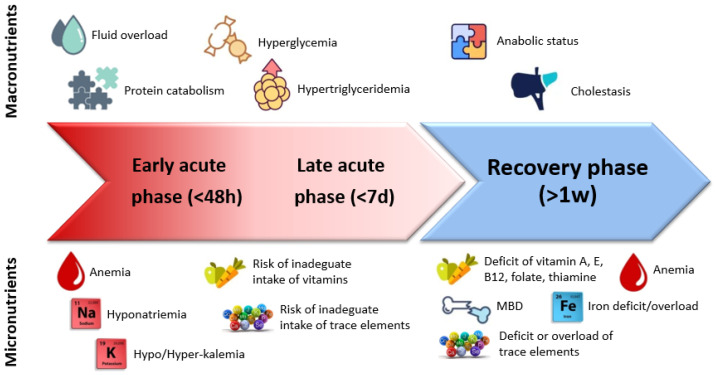
Timeline of metabolic response and main macro and micronutrient-related alterations in different phases of the disease (early acute, late acute, and recovery phase) [12,13,14,15,16,17,18,19,20,21,22,23].

Necrotizing enterocolitis (NEC) is the main gastrointestinal emergency in newborn infants, especially in preterms [1]. Although NEC incidence varies between centers [2,3], it is still 11–22% in very low birth weight infants (VLBWI) or extremely low birth weight infants (ELBWI), respectively [4,5], and has a significant mortality and morbidity (23.5% of NEC stage ≥II Bell and 50% of cases, respectively) [6]. Up to 40–50% of patients refractory to medical management (progressive clinical deterioration, multi organ failure) and all patients with perforated NEC require surgery [7]. These patients need extended periods of parenteral nutrition (PN) and are at high risk of nutrient imbalances and deficiencies (Figure 1), intestinal failure (IF), and consequent failure to thrive [6].

Additionally, these subjects develop Short Bowel Syndrome (SBS) in 42% of cases [8] and are at high risk of neurodevelopmental impairment at 18–22 months [9].

Bowel rest is the main therapeutic strategy in medical NEC, therefore PN is required after NEC onset regardless of the severity. PN is necessary during the acute phase as the only nutrition support and must be adapted to the rapid metabolic changes after NEC onset [10]. At the same time, it is essential during the recovery phase when enteral nutrition does not satisfy total nutrients’ requirement and especially if extensive bowel resection causes IF [11].

In this narrative review, we report the main evidence of the best parenteral nutrition strategy that clinicians should follow to provide the optimal intakes of nutrients, limiting the possible adverse effects and at the same time long-term deficiencies.

## 2. Before Surgery

In patients with NEC diagnosis, PN allows for bowel rest as an early measure to prevent disease progression, and it consists of glucose, amino acids, lipids, electrolytes, water, vitamins, and trace elements administered intravenously. PN should begin early after NEC onset to provide an adequate protein intake to allow repair of injured tissue [24,25], as well as glucose and lipids based on strict biochemical monitoring [12]. Furthermore, nutritional support and fluid administration at NEC diagnosis need to be adjusted according to several considerations. 

Infants affected by severe NEC (Bell stage > IIB) often have a multiorgan compromise, requiring frequent volume resuscitation and inotropic support due to poor vascular tone and capillary leak [13]. These, in turn, may lead to intestinal hypoperfusion with lactic acidosis, and electrolyte abnormalities. Therefore, in these patients, fluid volume intake must be carefully measured [14]. Acetate’s supplementation in PN is needed to partially correct acidosis [14]. Hyponatremia can also occur secondary to capillary leak and fluid-shift in the third spacing. For this reason, increasing requirements of sodium should be taken into consideration. Acidosis, reduced kidney function and intestinal ischemia can lead to hyperkaliemia in patients with progressive NEC. As a result, strict electrolytes monitoring and urine output is also required. 

## 3. At Surgery

Once the decision to proceed with surgery has been made [26], it is imperative to anticipate the physiologic impact of the stress response deriving from the surgical intervention itself. Operative intervention is often described as a controlled-induced trauma. Literature evidence are quite abundant for the adult population [27]. However, the metabolic rate and hormonal response to surgery differ between infants and adults, therefore it is not possible to adapt adult-based nutritional recommendations to the neonatal population. Several changes may occur in response to surgery stress. 

### 3.1. Variability in Metabolic Demand

Neonates with NEC were shown to have similar resting energy expenditure (REE) to healthy neonates [28] with a peak in REE of 60 kcal/g/day 4 h after surgery [29], returning to baseline, in the range of a basal metabolic range of 40–50 kcal/kg/day, within the next 12–24 h [30]. Nonetheless, the existing knowledge on the metabolic response to stress in infants is limited. It has been reported that oxygen consumption can be higher in infants undergoing surgery after the second day of life compared to that at birth [15] and a study by Powis et al. speculated that neonates, contrary to adults, divert the products of protein synthesis into tissue repair, reflecting a catabolic phase [31]. 

This is confirmed by the lack of growth commonly observed in infants with critical illnesses during the acute phase. Subsequently, gradual restoration of body protein and fat store follows the catabolic phase [32]. Therefore, clinicians should adapt energy intake through PN day by day according to the different phases of the metabolic stress response even if defining these phases in clinical practice may be challenging [16]. 

The recent position paper of ESPGHAN identifies three different phases of metabolic response in critically ill newborns: early acute phase (first 48 h); late acute (until 3–7 days) and recovery phase (after 1 week with the reoccurrence of anabolism) though preterms could have a shorter acute phase compared to term newborns [12]. Defining these phases clinically might be arduous and can be followed through biochemical parameters (C-reactive protein, variability of serum glucose, lactate, triglycerides, transthyretin, and urea) and clinical parameters (heart rate, respiratory rate, and blood pressure) though the early acute phase usually ends when the patient is stable and cardio-respiratory support might be reduced [12]. 

Neonatologists should carefully follow these phases, adapting energy intakes accordingly (Table 1).

### 3.2. Hyperglycemia

The increase of circulating catecholamines inhibits the insulin secretion with consequent hyperglycemia. This may be correlated to the degree of surgical stress and should be considered for the choice of fluids [36].

### 3.3. A positive Immunological Response

A positive immunological response activates the pro-inflammatory cytokines cascades: The immune system is activated to help tissue repair as well as to fight against post-operative infections. Pro-inflammatory cytokines stimulate gluconeogenesis (IL-1), hepatic lipogenis (IL-1, IL-6, TNF-alpha), and muscle proteolysis (TNF-alpha). These cytokines are balanced by anti-inflammatory mediators including Il-1ra and IL-10 and all of them have a direct impact on the metabolic demand, the fluid-shift in the third spacing and on the wound healing. The balance of pro- and anti-inflammatory response must be closely observed in these patients [15].

Defining the best timing to start PN in critical neonates is extremely varied [37]. Since PN should cover basic metabolic needs and allow for growth, children require relatively more macronutrients than adults. The current standard of pediatric intensive care is to meet these requirements early with a variable high dose of macronutrients in PN [38]. Based on large randomized controlled trials (RCTs) on ill adults that demonstrated the benefit of late PN [39], a pediatric randomized trial, the Early versus Late Parenteral Nutrition in the Pediatric Intensive Care Unit (PEPaNIC) trial was conducted, which showed that withholding PN during the first week of acute illness improved early outcomes (earlier discharge from the ICU and a lower infections rate) when compared to PN initiated during the first 24 h after injury [37]. A possible physiological explanation of this finding may be related to the activation of autophagy (a process essential for preservation of endogenous energy supply during critical illness). This mechanism is activated during fasting as well as during critical illness, however it is inhibited by early PN [40]. Despite the recent results of the PEPaNIC trial, which demonstrated that permissive underfeeding with PN is safe, recent European guidelines did not support a change in the current practice in preterm and suggest to start PN immediately to cover basic macronutrients needs [12]. However, recommendation advocates to begin PN at a minimal amount during the early acute phase (Table 1) [12,16,33,34,35,41], which is estimated to last 2–4 days in ill preterm and term neonates [32,41].

## 4. After Surgery

### 4.1. Fluid Balance

Frequent consequences of gastrointestinal surgery are the delay in bowel motility, which leads to ileus. Electrolyte abnormalities, gastrointestinal inflammation, and infections are the most common factors contributing to post-operative ileus. The choice of the type and doses of perioperative fluids may contribute to reduce the risk of post-operative ileus, however controversy still exists regarding the optimum fluid therapy [42]. In regard to fluid volume to be administered, fluid overload may cause post-operative respiratory failure due to fluid accumulation and decrease in tissue oxygenation, which has an adverse implication in wound healing. On the other hand, fluid volume restriction may lead to hypovolemia with consequent hypovolemic shock. Therefore, it is critical for fluid management to reach a balance between insufficient fluid resuscitation and excessive fluid administration [13]. In the immediate post-operative period, the total fluid administered should not exceed the maximum fluid intake recommended of 160–170 mL/kg/day [17], hence fluid administered by PN should be carefully restricted to avoid fluid overload but also macronutrients deficiency [13].

Although, in a retrospective study on newborns with a gestational age (GA) > of 31, the administration of a higher level of crystalloids seemed related to less postoperative complications, very little data have been published on the effect of fluid administration in newborns undergoing gastrointestinal surgery [43]. As far as the type of fluid is concerned, the current clinical practice suggests the use of crystalloids, consisting of isotonic saline or balanced electrolytes solutions (Ringer’s Solution), as the best choice to replace volume in the perioperative period as well as to improve perfusion and oxygenation and maintain a proper hydration. In addition, in case of a hypovolemic patient with metabolic acidosis, Ringer’s Lactate should be preferred because it leads to the excretion of H+, being poor in chloride [44]. A Cochrane review and metanalysis, though not considering newborns, suggested that the administration of colloids during resuscitation should be limited in those patients that might benefit from modest volume sparing and prolongation of intravascular expansion [45].

During the early post-surgical phase, patients are still at high risk for electrolyte abnormalities, especially infants with ileostomy or significant colon resection who can lose a great amount of sodium in their faeces. Hypokalaemia is also frequent due to increased urinary potassium loss as a result of both compensatory hyperaldosteronism stimulated by hyponatremia and massive use of diuretics [18]. Due to hepatic dysfunction, hypertriglyceridemia is frequent in the immediate post-surgical period [14].

During the recovery phase, with restoration of fecal output, water and electrolyte losses are often observed, in particular in the presence of enterostomy, hence it is important to carefully monitor fecal output and restore the losses [17]. Excessive fluid loss from stoma, defined as losses >30 mL/kg/day, might be frequently detected post-surgery requiring prompt diagnosis and treatment to avoid dehydration, electrolyte imbalance, and poor growth [46]. Prematurity, the inflammatory status, and antibiotics contribute to the high stoma losses that need to be replaced intravenously. The “Trust guideline for the management of stoma output in neonates and infants” of the Norfolk and Norwich University Hospitals suggested the use of 0.9% sodium chloride with 10 mmol of potassium/500 mL every 6 h (5 mL/kg 6 hourly) to replace losses higher than 20 mL/kg/day and to frequently monitor serum electrolytes, blood gas, and weight [47].

In conclusion, careful monitoring of fluid and electrolyte balance is necessary and a tight control of indicator of hydration should be done both in the post-operative phase and in the recovery phase (Figure 1), with strict monitoring of body weight, urine output, fluid balance, blood and urine electrolyte concentration, acid-base status, haematocrit, and blood urea nitrogen [17].

### 4.2. Energy and Macronutrients Needs

After surgery and during the recovery phase, higher energy and fat intakes seem to be necessary to synthetize new proteins for tissues repair, to reduce fat oxidation, and facilitate lipogenesis [32,48]. As recently suggested, the energy needs during the recovery phase should increase up to 90–120 kcal/kg/day [16].

All these considerations result from studies of critical ill newborns without strong focus on NEC patients: further studies are needed to completely understand the caloric requirement in the special population of infant after surgical NEC (Table 1).

The caloric needs for total PN are provided by carbohydrates and lipids. Protein should not be used as a source of calories, since the catabolism of protein to produce energy is an uneconomic metabolic process compared to the oxidation of carbohydrate and fat, which produces more energy at a lower metabolic cost [13]; therefore, lipid should be 30–50% of nonprotein calories and energy from fat and glucose >25 kcal/g of protein [16]. 

#### 4.2.1. Glucose

Glucose is the primary energy substrate in PN, covering 50–70% of non-protein calories. Infants with established NEC have a high prevalence of hyperglycemia and a worse outcome if hyperglycemic [19]: increased levels of stress hormones and inflammatory cytokines secondary to NEC onset-induced transient insulin resistance that causes hyperglycemia, especially during the acute phase [49]. Furthermore, preterm infants are particularly susceptible to hyperglycemia due to immature regulatory mechanisms and decreased insulin production [50]. It is recommended that newborns <28 days of age, who have an episode of acute illness, should temporarily receive the carbohydrate supply of the first day of life (5.8–11.5 g/kg/day for preterm infant; 3.6–7.2 g/kg/day for term infants) and then increased, guided by blood glucose level [51]. It is also recommended to begin insulin therapy at 0.05 IU/kg/h just in case of repeated blood glucose levels >10 mmol/L (180 mg/dL) despite a reasonable adaptation of the glucose infusion rate (4–6 g/kg/day in term or preterm infants, respectively) [16]. The maximum glucose infusion rate recommended by ESPGHAN is 17.3 g/kg/day [51]. Jones et al. demonstrated that in surgical newborns when glucose intake exceeds 18 g/kg/day, plasma insulin increases, triggering hepatic lipogenesis and steatosis contributing to PNALD [35]. However, the role of high carbohydrate intake in the pathogenesis of PNALD is controversial [52]: Jacobsen et al. showed that a low-fat, high-carbohydrate PN regimen (17.2 g/kg/day glucose intake) may be used in reversing liver disease in PN-dependent infants without compromising growth [53]. 

In conclusion, after surgery, glucose should be provided and increased along with a tight monitoring to keep plasma glucose level <10 mmol/L. During the recovery phase, if expectation of long-term PN is concrete, an excessive glucose intake (>17.3 g/kg/day) is not recommended.

#### 4.2.2. Amino Acids

Adequate protein intake is essential for preventing catabolism and promoting growth, then PN should provide adequate protein to maintain a positive nitrogen balance, improve weight gain, and to allow repair of injured tissue [54]. 

A pilot study showed that the whole protein turn-over in neonates with proven NEC is comparable to the value in stable neonates, and that neonates with NEC may divert the products of protein synthesis from growth to tissue repair [28]. A prospective study compared a high dose of protein (median daily dose 3.87 g/kg) versus a low dose of protein (median daily dose 2.80 g/kg) during the first week after surgery for NEC, showing no difference in body growth with the exception for head growth (increased in the first group), however, of significant concern, non-survivors received less protein than survivors [10]. Regarding the composition of aminoacidic solutions, few data are available:

- Glutamine is the most abundant free amino acid in the body and it is the preferred respiratory fuel for rapidly proliferating cells. In case of metabolic stress, the endogenous production of glutamine could be insufficient, becoming a “conditionally essential amino acid”. Meta-analyses do not provide evidence that glutamine supplementation gives advantage for preterm infants [55]. Furthermore, studies focusing on surgical newborns also showed no benefit from glutamine supplementation during PN [56,57].
- Taurine (synthesized from methionine and cysteine) deficiency may increase glyco-conjugates of bile acids resulting in cholestasis [58]. Studies showed that prolonged PN with a taurine-free parenteral solution resulted in reduced plasma taurine levels [59]. Although the cause of neonatal cholestasis and PNALD is probably multifactorial, there are few data indicating that adequate taurine may prevent cholestasis, in particular in patients with NEC [60]. The right dose of taurine supplementation is not yet known [58]: Spenser et al. demonstrated some benefits from taurine integration with a dose ranging from 6 to 21.6 mg/kg/day depending on the total protein delivery and formulation [60].
- Arginine supplementation was well tolerated and resulted in a significant decrease in the incidence of NEC [61,62], however no studies investigated the advantage of arginine supplementation after NEC onset.

In conclusion, we should take into account that some non-essential amino acids could become conditionally essential in stressful circumstances, especially in preterm newborns [60,63]. Moreover, even though early amino acid intake is related to better outcomes [64,65], we still do not know how much protein is too much as we do not have a reliable marker of amino acid toxicity. Indeed, higher amino acid intakes (4 g/kg/day) might result in excess levels of some amino acids [66] and in temporarily lower Mental Development Index scores at 18 months [67]. We know that the urea level positively correlates with amino acid intakes [68,69]. Nonetheless, high urea levels might not always reflect amino acid intolerance, and it just might be a sign of appropriate utilization of amino acid, as it happens during the fetal life [70]. 

**Figure 2:**
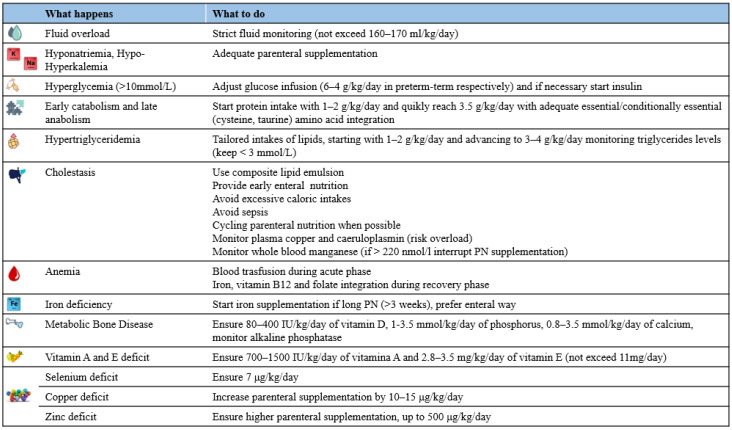
Summary of macro and micronutrients’ abnormalities after NEC) surgery and suggested action [12,14,16,17,18,20,21,22,49,51,58,71,72,73,74,75,76,77,78,79,80,81,82,83,84].

Therefore, the ideal amino acid requirement of neonate after NEC is still controversial, however it should be 1–2 g/kg/day, the first day after surgery and quickly increase up to 2.5–3.5 g/kg/day, accompanied by non-protein intakes >65 kcal/kg/day [16,58,71] (Figure 2).

#### 4.2.3. Lipids

Intravenous lipid emulsions (ILEs) are fundamental components of pediatric PN as they represent a high-caloric energetic non-carbohydrate source with low osmolarity [20]. After surgery for NEC, it is still debated when to begin lipid supplementation, which is the best lipid source and how much lipid intake should be provided to preterm newborns. Additionally, the maximum amount of lipids that can be safely given in premature infants is currently unknown [20], however PN free of ILEs may lead to essential fatty acids (EFA) deficiency within a few days [85]. In the recent position paper on the critically ill preterm newborn, though not specifically considering patients with NEC after surgery, the minimum energy to provide the basal metabolic rate is recommended [16]. It is also known that a linoleic acid intake of 0.25 g/kg/day must be provided in preterm infants to avoid EFA deficiency [20], which can be achieved with 0.5 g/kg/day or 1 g/kg/day depending on the source of lipids. 

Patients with NEC are at risk of hypertriglyceridemia as reduced hepatic perfusion can lead to poor fat metabolism [14]. In addition, excessive carbohydrate intakes can enhance lipogenesis and contribute to hypertriglyceridemia. Afterwards, in the stable phase, studies focusing on preterm infants suggest parenteral lipid intake up to 3.0–4.0 g/kg/day [85]. Although there are no evident benefits of a gradual increase of lipid intakes, beginning with a minimum amount to provide EFA (1 g/kg/day) and then increasing to 3–4 g/kg/day, maintaining a triglyceride level <3 mmol/L seems a reasonable approach [20]. 

Another aspect is that after NEC surgery, prolonged PN use (>21 days) may be required and this is a significant risk factor for PNALD [72,86], which is defined as a bilirubin level of 2 mg/dL (34.2 μmol/L) or greater with or without raised liver enzymes in the absence of other causes [73]. An odds ratio was reported for developing cholestasis of 13.1 in preterm newborns <750 g of birth weight, a value that is reduced to 2.8 in the preterm of 1000–1500 g of birth weight [87], however a value, still, of 3.3 was reported in small for gestational age neonates compared to appropriate ones [88]. 

Some modifications can be made to reduce the risk for liver injury when prolonged PN is required, such as avoidance of PN overfeeding and optimization of other macronutrients intake [74], maintaining oral or enteral intake (whenever possible) [74], cycling lipid and PN infusions when possible [75], minimizing recurrent episodes of sepsis [76], limiting the dose of soybean-based or use of composite lipid emulsion [20]. Soy-based lipid emulsions (SO-LE) have been the principal source of lipids for PN. It is known, from animal studies, that high levels of omega-6 Long Chain Triglycerides can trigger inflammation and oxidation resulting in liver damage [89]. Soy emulsion are rich in phytosterols, that by the enteral way would be only poorly absorbed, however in the parenteral route instead may lead to liver damage. In preterm infants, especially in those with cholestasis, phytosterols have a longer half-life [90,91,92], therefore exposing these subjects to an increased risk of liver injury. 

However, Savini et al. [93] did not report a lower cholestasis rate in preterm infants by using lipid emulsion with lower phytosterol content. To reduce proinflammatory factors, other lipid emulsions have been studied and produced. The term “mixed oil lipid emulsions” (MO-LE) is used in the literature as a very general term for any emulsion not comprised of a single-source lipid [72]. European studies have demonstrated that mixed fatty acid emulsions, which contain soybean, fish, olive, and coconut oils, are associated with improved liver function, decreased markers of inflammation and oxidative injury, and increased antioxidant activity [94]. Fish oil lipid emulsion (FO-LE) instead is pure fish oil containing high concentrations of eicosapentaenoic acid (19%) and docosahexaenoic acid (12%) and low concentrations of linoleic acid (4.4%), alpha linoleic acid (1.8%), and arachidonic acid (1–4%) [72]. Studies have provided evidence that when 1 g/kg/day of exclusive FO-LE is substituted for SO-LE, direct hyperbilirubinemia is more likely to resolve, and the incidences of death and transplant may be reduced [95]. FO-LE did not resolve PNALD, however it did stop further progression of the disease as assessed by direct bilirubin levels [96].

Nonetheless, controversies exist on the real benefit of FO-LE on the incidence of cholestasis. In a metanalysis by Park et al. [97], the incidence of PNALD was not significantly reduced in newborns receiving a lipid emulsion containing fish oil compared to a soybean one. On the contrary, two recent metanalysis found a reduced risk of cholestasis in patients receiving a mixture of lipids containing fish oil versus pure soybean-based or olive-oil-based emulsion [98,99]. The most recent Cochrane meta-analysis [73] did not find a significant difference in the incidence of PNALD between FO-LEs and all non-fish oil LEs. 

However, especially in newborns at risk of developing cholestasis (preterm, prolonged bowel rest, sepsis, SBS), a lower level of proinflammatory cytokines was found in those receiving FO-LE compared to the soybean one [100].

Recent guidelines on lipid intakes report that for “PN lasting longer than a few days, pure SO-LE should no longer be used, and composite intravenous lipid emulsions with or without fish oil should be the first choice” [20].

In conclusion after surgery for NEC, a basal intake of lipids (1–2 g/kg/day) must be provided and gradually increased to 3–4 g/kg/day, keeping triglycerides level <3 mmol/L, starting with composite lipid emulsion (Figure 2).

### 4.3. Micronutrients

#### 4.3.1. Vitamins and Electrolytes

Electrolytes and vitamins with trace elements are structurally important as cofactors or as components of enzymes, and provision of adequate supplies are important for the growing neonate. However, for many of these, the precise requirements are still debated [13]. During the full PN support but also during the transition to full enteral feeding, neonates with NEC are at risk of developing micronutrient deficiency and this is mainly true in cases that evolve in SBS and IF [77,101]. There is a positive relationship between the risk of developing micronutrient deficiency and length of PN [101]. Up to date, few studies have focused on micronutrient requirements, in particular during the early period after surgery.

We also have to remember that electrolytes’ plasma levels could be found in range despite a reduced body content, therefore their levels in urine should be checked routinely. In particular, urinary sodium should be kept >20–30 mEq/L to meet the elevated newborn’s requirements for growth [102,103].

Regarding vitamins, to the best of our knowledge, no studies have evaluated the metabolism of two fat soluble vitamins, A and E, after surgery for NEC. However, patients with intestinal failure have a high risk of vitamin A and E deficiency, frequently asymptomatic, during prolonged PN [104], as well as during transition to full enteral nutrition and when full enteral nutrition is reached [101,105,106]. Therefore, parenteral preparation should be supplemented with these vitamins as soon as possible after surgery, as suggested by the current ESPGHAN and ASPEN recommendations: For vitamin A 700–1500 IU/kg/day in preterm newborns and 150–300 IU/kg/day in term infants; for vitamin E 2.8–3.5 mg/kg/day not exceeding 11 mg/day [21,107,108].

The other important fat-soluble vitamin whose metabolism could be changed by intestinal surgery is vitamin D, which also influences the calcium-phosphorus balance and bone health. Bowel resection reduces vitamin D absorption and cholestasis can worsen it [22,109]. Additionally, calcium and phosphorus deficiencies are frequent after NEC [110], in particular surgical NEC, secondary to malabsorption and inadequate intakes [77,111]. Hypocalcemia results in secondary hyperparathyroidism, which contributes to bone resorption [112]. This is confirmed by the fact that patients with NEC have higher bone resorption markers than infants without NEC [113], as well as an increased risk of metabolic bone disease (MBD) and rickets [23]. Preterm infants are already at risk of MBD, which occurs in 23% in VLBWI or even 55% in those <1000 g of birth weight [114]. Several factors are related to the high incidence of MBD in preterms: insufficient mineral and caloric intakes [78,115], poor motor activity [116,117], use of drugs [118], PN lasting more than 3–4 weeks [119], or even only more than 2 weeks [120,121]. Controlling phosphorus and alkaline phosphatases (ALP) may be useful to monitor MBD; while plasma calcium levels are kept normal by PTH, low phosphorus and high ALP correlated with reduced bone density [122], and those preterm with an elevated ALP > 1200 IU/L had reduced height at 18 months and 12 years of age [123]. Another important point is that preterm newborns are at risk of refeeding syndrome [124] when high glucose and amino acids intakes are provided with low phosphorus and potassium supply, therefore a thorough control of those electrolytes must be performed especially in the recovery phase [16].

As for other fat-soluble vitamins, no specific recommendation on the intake of vitamin D, calcium, and phosphorus exists for infants with NEC. However, due to a high risk of deficiency for this special population, we reiterate the current ESPGHAN recommendation [21,107,108]:

- A total of 200–1000 IU/day (or 80–400 IU/kg/day) of vitamin D for preterm infants and 400 IU/day (or 40–150 IU/kg/day) for term infants up to 12 months of age;
- A minimum of 0.8 mmol/kg/day up to 3.5 mmol/kg/day of calcium in preterm infants;
- A minimum of 1 mmol/kg/day up to 3.5 mmol/kg/day of phosphorus in preterm infants.

We also suggest to periodically monitor vitamin D deficiency and in case of a 25 OH-vitamin D blood level under 50 nmol/L to provide additional supplementation, knowing that parenteral vitamin D therapy could be effective and safe [125].

No different suggestion exists for vitamin K intake in patients with NEC, accordingly, a general recommendation of 10 μg/kg/day in preterm infants is valid even after NEC [21].

Focusing on water-soluble vitamins, resection of ileum, in particular the terminal tract, causes a decrease of vitamin B12 absorption [126,127]. Therefore, parenteral supplementation is essential in this cohort of patients and the current recommendation avoids deficiency [21]. Thiamine and folate are two other water-soluble vitamins at risk for deficiency in patients on long-term PN secondary to intestinal surgery, even though available studies do not focus on a post-surgery phase of NEC [128].

In conclusion, after NEC surgery newborns have a high risk for vitamin deficiency due to both intestinal malabsorption and long PN, we suggest beginning parenteral supplementation as soon as possible after surgery and to plan a periodical monitoring specially if wide resection or enterostomy were made (Figure 2).

#### 4.3.2. Trace Elements

The appropriate provision of trace elements in PN is essential to improve and maintain nutrition status and prevent complications or deficiency.

No studies investigated the metabolism of trace elements during the acute phase after surgery for NEC. More evidence exists for long-term PN, in particular for cases of NEC evolved into SBS and IF.

- Deficiency of selenium may occur during the total PN [22,104] and seems to improve after restoration of enteral feeding [101,106]. For the surgical infant, additional supplementation should be considered based on the estimation of fecal losses, up to 7 μg/kg/day [79,129].
- Manganese should be supplied at a dose of no more than 1 μg/kg/day, however with blood levels regularly monitored as there is high risk of accumulation with neurotoxicity [130,131]. This risk is increased in neonates due to the evidence of significant quantities of Mn in neonatal PN as a contaminant [132,133]. Patients who develop PNALD are at a higher risk of Mn accumulation [79,134]: In case of PNALD whole blood manganese should be determined and if >220 nmol/L, parenteral supplementation should be discontinued [79,80].
- Anemia often occurs during the acute phase due to several causes, especially bleeding and blood test monitoring. During this phase, no evidence suggests iron deficiency since multiple transfusions are needed and might give iron support. However, prolonged PN (particularly > 3 weeks) without iron supplementation may induce iron deficiency and anemia [22,101]. For this reason, it is important to plan iron supplementation that should preferentially be given enterally rather than with intermittent infusions due to adverse drug reactions, in particular anaphylaxis [80]. If patients develop SBS, the incidence of anemia is controversial [22,77,101,105,106] and it can be explained by blood draws, iron, or vitamin B12 deficiency, recurrent infection and intestinal anastomotic ulcers that can cause refractory anemia [77,81]. Therefore, the iron status, comprising serum ferritin and hemoglobin, should be regularly monitored in patients on long-term PN in order to prevent iron deficiency and iron overload [80].
- Salivary, gastric, pancreatic, and intestinal juices contain a significant amount of zinc that normally is reabsorbed in the proximal small intestine, more specifically in the distal duodenum and proximal jejunum [135]. Newborns with elevated enteral fluid losses (diarrhea, steatorrhea, or stoma losses) are at a high risk for zinc deficiency [136]. Many case series document zinc deficiency in newborns with bowel damage, especially with jejunostomy or ileostomy [22,79,101,106,137]. Symptoms of zinc deficiency are well known, ranging from weight loss, failure to thrive, periorificial dermatitis, glossitis, and increased susceptibility to infections [138,139,140,141,142,143]. Intravenous zinc supplementation of 400 to 500 μg/kg/day in preterm infants is recommended, however no specific recommendations in infants with small intestinal stoma are available [80]. A monocentric study by D’Aniello et al. showed that a zinc deficit is prevented in newborns with a small bowel stoma if supplementation is 500 μg/kg/day parenterally [82]. Therefore, higher parenteral zinc supplementation should be planned early in infants after surgical NEC with jejunostomy or ileostomy.
- Similarly, copper deficiency can be found in infants with jejunostomy or ileostomy or long-term PN [22,77,106,142]. Copper is primarily absorbed via the small intestine and patients who have increased copper losses through stool or ostomy require additional supplementation in PN solutions by 10–15 μg/kg [137]. However, there is no agreement on the best parenteral copper dose: The American Society for Clinical Nutrition and the ASPEN recommend 20 μg/kg/day [107,108], while the ESPGHAN guideline recommends 40 μg/kg/day in preterm infants and 20 μg/kg/day in term infants [80]. Additionally, there are no specific indications for surgical patients. Adler et al. found that 20 μg/kg/day of copper in PN of neonates with ostomies is insufficient to prevent Cu deficiency [83]. Moreover, the predominant pathway of copper excretion is through bile and it remains common practice to reduce or eliminate copper in the PN solutions of infants with PNALD because of the risk of hepatic toxicity.

However, this practice may determine copper deficiency and recent data [144,145] suggest that cholestasis does not appear to impair copper excretion, with new strategies emerging, such as the intermittent parenteral copper supplementation [84]. In conclusion, it is important to monitor plasma copper and ceruloplasmin, especially in the case of PNALD or gastrointestinal fluid losses infants [80]. Further studies are warranted to determine an optimal dosage of parenteral Cu in surgical infants.

Regarding the other trace elements, no studies have focused on the unique population of infants after surgical NEC. We can give some advice considering that this cohort of patients are at high risk for SBS and IF. Molybdenum is a cofactor of enzymes implicated in purine, pyrimidine and amino acid metabolism. Sievers et al. [146] showed negative molybdenum balance in infants with ostomy, not specifically for NEC, that may indicate an increased risk for molybdenum deficiency. However, no other evidence supports altered Molybdenum physiology in the surgical infant [79]. 

In conclusion, it is important to know that infants with NEC might need long-term PN with a high risk of imbalance of trace element, especially a deficiency of selenium, iron, zinc, copper, molybdenum, and iodine, and an overload of manganese, copper, and iron. 

Close blood monitoring of these elements, together with other micronutriens and also macronutriens, should be planned to adjust the parenteral intake (Figure 1 and Figure 2).

## 5. Conclusions

Parenteral nutrition is essential in the management of patients with NEC, both at time of diagnosis and after surgery. The provision of macronutrients and micronutrients should be planned according to the current knowledge and international recommendations. 

Micronutrients and macronutrients deficiency and overload should be routinely monitored. In addition, clinical and biochemical signs of PN and surgery-related morbidities, including IF, PNALD, MBD, and growth failure, should be carefully assessed. Though NEC results in SBS in 42% of cases, few studies have focused on this special population; therefore, further studies are warranted to improve the nutritional approach and the management of long-term consequences in these newborns. 

## Figures and Tables

**Table 1:** Energy requirements in parenteral nutrition (kcal/kg/day) during the different phases of disease in newborns, according to different authors.

Studies	Early Acute	Late Acute	Recovery
Moltu et al., 2021 [12]	40–55	60–80	90–120
Joosten et al., 2018 [16]	45–55	60–65	90–120
Feferbaum et al., 2010 [33]	49.4 +/− 13.1	/	68.3 +/− 10.9
Bauer et al., 2002 [34]	58 +/− 3	55 +/− 2	50 +/− 2
Jones et al., 1993 [35]	40.1–60.5 for 4–7 days post-surgery		
